# Hyperhomocysteinemia and Mortality after Coronary Artery Bypass Grafting

**DOI:** 10.1371/journal.pone.0000083

**Published:** 2006-12-20

**Authors:** Domenico Girelli, Nicola Martinelli, Oliviero Olivieri, Francesca Pizzolo, Simonetta Friso, Giovanni Faccini, Claudia Bozzini, Ilaria Tenuti, Valentina Lotto, Giuliano Villa, Patrizia Guarini, Elisabetta Trabetti, Pier Franco Pignatti, Alessandro Mazzucco, Roberto Corrocher

**Affiliations:** 1 Department of Clinical and Experimental Medicine, University of Verona Verona, Italy; 2 Institutes of Clinical Chemistry, University of Verona Verona, Italy; 3 Section of Biology and Genetics, University of Verona Verona, Italy; 4 Cardiovascular Surgery, University of Verona Verona, Italy; Baylor College of Medicine, United States of America

## Abstract

**Background:**

The independent prognostic impact, as well as the possible causal role, of hyperhomocysteinemia (HHcy) in coronary artery disease (CAD) is controversial. No previous study specifically has addressed the relationship between HHcy and mortality after coronary artery bypass grafting (CABG) surgery. The aim of this study is to evaluate the prognostic impact of HHcy after CABG surgery.

**Methodology and Principal Findings:**

We prospectively followed 350 patients who underwent elective CABG between May 1996 and May 1999. At baseline, fasting total homocysteine (tHcy) levels were measured in all participants, and a post-methionine loading (PML) test was performed in 77.7% of them (n = 272). After a median follow-up of 58 months, 33 patients (9.4%) had died, 25 because of cardiovascular events. HHcy, defined by levels higher than the 90^th^ percentile (25.2 µmol/L) of the population's distribution, was significantly associated to total and cardiovascular mortality (*P* = 0.018 [log-rank test 5.57]; *P* = 0.002 [log-rank test 9.76], respectively). The PML test had no prognostic value. After multiple adjustment for other univariate predictors by Cox regression, including statin therapy (the most powerful predictor in uni-/multivariate analyses), high-sensitivity C Reactive Protein (hs-CRP) levels, and all known major genetic (*MTHFR 677C→T* polymorphism) and non-genetic (B-group vitamin status and renal function) tHcy determinants, HHcy remained an independent prognostic factor for mortality (HRs: 5.02, 95% CIs 1.88 to 13.42, *P* = 0.001).

**Conclusions:**

HHcy is an important prognostic marker after CABG, independent of modern drug therapy and biomarkers.

## Introduction

Homocysteine is a sulphur-containing amino acid formed as a by-product of methyl-transfer reactions in methionine metabolism [1]. Since proposed in 1969 by McCully [2], the association between serum total homocysteine (tHcy) and coronary artery disease (CAD) has been strongly corroborated by recent meta-analyses of numerous epidemiological studies [3]. Nevertheless, the independent prognostic impact of tHcy, as well as its causal role in CAD pathogenesis, remain controversial. Pieces of evidence in favor include basic research studies on animal models indicating that Hcy causes endothelial dysfunction/damage, lipid peroxidation, and accelerated thrombosis [reviewed in 4]. Studies on the common *677C→T* polymorphism of the Methylentetrahydrofolate reductase (*MTHFR*) gene, known to be associated with a moderate elevation of tHcy, support a causal link between Hcy and atherosclerosis through the so-called “Mendelian randomization” phenomenon [5], though some Authors recently disagreed [6]. Critics also emphasize the lack of evidence, from the randomized controlled trials published so far [7]–[10], that lowering tHcy by folic acid-based multivitamin therapy could prevent “hard” vascular outcomes. Finally, it is frequently underscored that the stronger associations between Hcy and vascular risk have been found in cross-sectional or case-control studies rather than in prospective studies [3], [11], which are generally considered superior in supporting causation. Indeed, results from epidemiological studies on cardiovascular patients are often weakened by the wide clinical heterogeneity of the patients enrolled, as well as that of the endpoint considered.

We endeavored to further evaluate tHcy as secondary risk predictor by studying prospectively a selected homogeneous population of patients undergoing coronary artery by pass grafting (CABG), which remains a cornerstone of CAD therapy even in the percutaneous coronary intervention (PCI) era [12]. Total and cardiovascular mortality were considered as the endpoints.

## Methods

### Study population

The Verona Heart Project is a study aimed to search for new risk factors for CAD in a population of subjects with objective angiographic documentation of their coronary vessels. Details about the enrolment criteria have been described elsewhere [13]. In the present study we used data from a total of 353 consecutive adult patients of both sexes, who underwent to CABG at the Cardiosurgery Unit of Verona between May 1996 and May 1999. Angiographic CAD was assessed by two cardiologists unaware that the patients were to be included in the study. The majority of patients (93.4%) had severe multi-vessel CAD (stenosis≥50% of the vessel diameter in two or three of the main coronary arteries). All participants came from the same geographical area (Northern Italy), with a similar socio-economic background. They were enrolled at scheduled ambulatory evaluation few days before elective CABG surgery. At that time, blood sampling and a complete clinical history was collected, including the assessment of classical cardiovascular risk factors. The diagnosis of previous myocardial infarction was based on a thorough review of the medical history, previous ECG, and enzyme documentation, and/or based on the finding of typical sequelae of infarction on ventricular angiography. Left ventricular ejection fraction (LVEF) was estimated by ventriculography and/or echocardiography. To avoid any bias in tHcy measurement, we enrolled only subjects who were not treated with vitamin supplementation or drugs interfering with tHcy levels (i.e., anticonvulsivants, methotrexate and penicillamine) [13]. Similarly, none of the enrolled subjects had any major systemic acute illness, including myocardial infarction (MI), in the preceding 3 months. The study was approved by the Ethical Committee of our Hospital. Informed consent was obtained from all the patients after a full explanation of the study.

### Assessment of outcome

Subjects were followed until death or May 2004. Study subjects status was determined by search of the national population register, and by an ambulatory or telephone survey. Ascertainment of mortality status was complete (100 percent). The date of death was obtained from the National Population Register. The causes of death were obtained from death certificates kept at the Italian Institute of Statistics (ISTAT). Death from cardiovascular causes was defined as death caused by CAD, heart failure, peripheral vascular disease, or cerebrovascular disease. Three subjects died peri-operatively (during hospitalization and/or within one month from surgery), so that they were excluded from the survival analysis.

### Laboratory testing

Samples of venous blood were drawn from each subject after an overnight fast. Serum lipids, other routine biochemical parameters including creatinine, as well as plasma folate, vitamin B12, and the *MTHFR 677C→T* polymorphism were determined as previously described [13]. The four variable version of the Modification of Diet in Renal Disease (MDRD) equation was used to estimate the glomerular filtration rate (GFR) from serum creatinine levels. Vitamin B6 was measured by high-pressure liquid chromatography (HPLC) with fluorometric detection [14]. High-sensitivity C-reactive protein (hs-CRP) was measured by particle-enhanced nephelometric immunoassay (Dade-Behring Inc., Newark, DE). For tHcy (which refers to the sum of homocysteine, homocystine, and homocysteine-cysteine mixed disulfide, free and protein bound), blood was collected into EDTA-containing vacuum tubes and kept on ice and in the dark; plasma was separated within 90 minutes; tHcy levels were determined by HPLC with fluorometric detection, as described [13], [15]. In 272 subjects, after the first blood sampling, a standardized methionine-loading test was performed by administering orally L-methionine (100 mg/kg) mixed with 200 ml of orange juice, together with a standardized low-protein breakfast; blood was then collected 6 hours later for the determination of the post-methionine loading (PML) tHcy level.

### Statistics

All the calculations were performed with SSPS 13.0 statistical package (SPSS Inc., Chicago, IL). Distributions of continuous variables in groups were expressed as means±standard deviation. Logarithmic transformation was performed on all skewed variables, including tHcy, folate, vitamin B12, vitamin B6 and hs-CRP. Hence, geometric means with 95% confidence intervals (CIs) are given for these variables. Quantitative data were assessed using the Student's t-test, or by ANOVA with Tukey's post-hoc test when appropriate. Associations between qualitative variables were analyzed with the χ^2^ test. Correlations between quantitative variables were assessed using Pearson's correlation test. Significant associations between tHcy levels and other continuous or categorical variables, were assessed by multiple regression models estimating R^2^ and standardized β-coefficients. Categorical variables were used after transformation into binary variables (i.e. for *MTHFR 677C→T* polymorphism we considered homozygosity versus non-homozygosity for the T allele). Hyperhomocysteinemia (HHcy) was defined by tHcy levels higher than the 90^th^ percentile of population's distribution, i.e. ≥25.2 µmol/l. Survival was assessed by the Kaplan-Meier method (log-rank statistic) and Cox regression. Kaplan-Meier methods were used for survival plots. Multivariate Cox proportional hazards analyses were performed considering sex, age, other tHcy determinants (MTHFR genotype, B-vitamin levels, GFR), and all predictors of mortality at univariate analyses, including drug therapy at discharge. Final models were obtained by a backward stepwise logistic regression approach, with *P* = 0.10 as the critical value for entering/excluding variables in the model. Hazard ratios (HRs) and 95% CIs are reported with two-tailed probability values. A *P* value less than 0.05 was used to indicate statistical significance.

## Results

### Characteristics of the patients

Table 1 (left column) shows the complete clinical and biochemical characteristics at baseline of the patient's population (n = 350) after exclusion of peri-operative deaths (n = 3; tHcy values: 18.6, 11.7, and 11.4 µmol/l, respectively). The mean age of patients was 60.2±8.9 years, 82.9% were males, 61.8% had had a previous MI, 93.4% had multi-vessel disease. They had a substantial burden of traditional cardiovascular risk factors, such as smoking (65.6%), hypertension (66.2%), and so on. Patients with HHcy were similar to the others in age, sex, degree of CAD, history of MI before CABG, drug therapy at discharge, and most other traditional risk factors (Table 1, right columns). On the other hand, HHcy patients had decreased GFR and B-vitamin levels, while hs-CRP levels and homozygosity for the *T* allele of *MTHFR* were increased. The PML increase of tHcy over basal levels (Δ PML tHcy) was similar in the two groups.

**Table 1:**
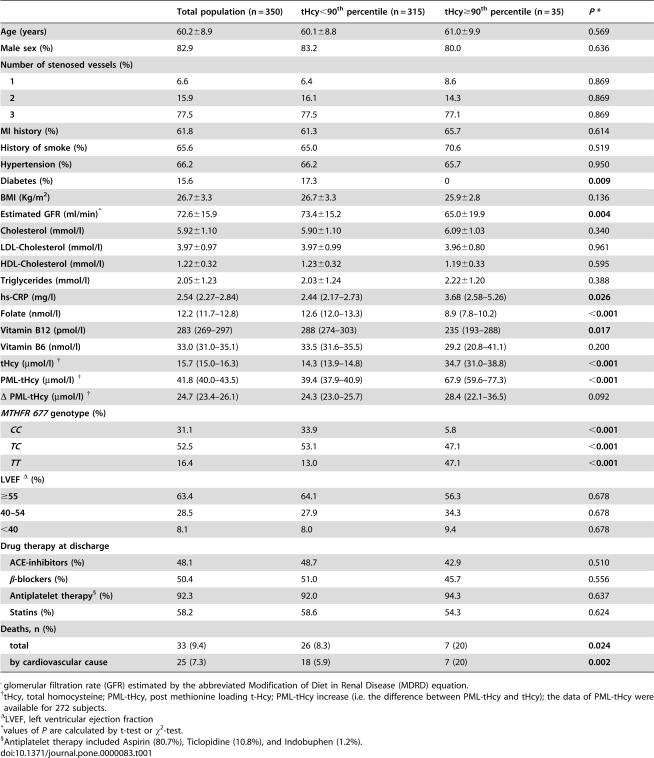
Clinical and laboratory characteristics of patients by baseline tHcy (90^th^ percentile = 25.2 µmol/l).

	Total population (n = 350)	tHcy<90^th^ percentile (n = 315)	tHcy≥90^th^ percentile (n = 35)	*P* *
**Age (years)**	60.2±8.9	60.1±8.8	61.0±9.9	0.569
**Male sex (%)**	82.9	83.2	80.0	0.636
**Number of stenosed vessels (%)**				
**1**	6.6	6.4	8.6	0.869
**2**	15.9	16.1	14.3	0.869
**3**	77.5	77.5	77.1	0.869
**MI history (%)**	61.8	61.3	65.7	0.614
**History of smoke (%)**	65.6	65.0	70.6	0.519
**Hypertension (%)**	66.2	66.2	65.7	0.950
**Diabetes (%)**	15.6	17.3	0	**0.009**
**BMI (Kg/m^2^)**	26.7±3.3	26.7±3.3	25.9±2.8	0.136
**Estimated GFR (ml/min)** ^	72.6±15.9	73.4±15.2	65.0±19.9	**0.004**
**Cholesterol (mmol/l)**	5.92±1.10	5.90±1.10	6.09±1.03	0.340
**LDL-Cholesterol (mmol/l)**	3.97±0.97	3.97±0.99	3.96±0.80	0.961
**HDL-Cholesterol (mmol/l)**	1.22±0.32	1.23±0.32	1.19±0.33	0.595
**Triglycerides (mmol/l)**	2.05±1.23	2.03±1.24	2.22±1.20	0.388
**hs-CRP (mg/l)**	2.54 (2.27–2.84)	2.44 (2.17–2.73)	3.68 (2.58–5.26)	**0.026**
**Folate (nmol/l)**	12.2 (11.7–12.8)	12.6 (12.0–13.3)	8.9 (7.8–10.2)	**<0.001**
**Vitamin B12 (pmol/l)**	283 (269–297)	288 (274–303)	235 (193–288)	**0.017**
**Vitamin B6 (nmol/l)**	33.0 (31.0–35.1)	33.5 (31.6–35.5)	29.2 (20.8–41.1)	0.200
**tHcy (µmol/l)** †	15.7 (15.0–16.3)	14.3 (13.9–14.8)	34.7 (31.0–38.8)	**<0.001**
**PML-tHcy (µmol/l)** †	41.8 (40.0–43.5)	39.4 (37.9–40.9)	67.9 (59.6–77.3)	**<0.001**
*Δ* **PML-tHcy (µmol/l)** †	24.7 (23.4–26.1)	24.3 (23.0–25.7)	28.4 (22.1–36.5)	0.092
***MTHFR 677*** ** genotype (%)**				
***CC***	31.1	33.9	5.8	**<0.001**
***TC***	52.5	53.1	47.1	**<0.001**
***TT***	16.4	13.0	47.1	**<0.001**
**LVEF** Δ **(%)**				
**≥55**	63.4	64.1	56.3	0.678
**40–54**	28.5	27.9	34.3	0.678
**<40**	8.1	8.0	9.4	0.678
**Drug therapy at discharge**				
**ACE-inhibitors (%)**	48.1	48.7	42.9	0.510
**β-blockers (%)**	50.4	51.0	45.7	0.556
**Antiplatelet therapy** § **(%)**	92.3	92.0	94.3	0.637
**Statins (%)**	58.2	58.6	54.3	0.624
**Deaths, n (%)**				
**total**	33 (9.4)	26 (8.3)	7 (20)	**0.024**
**by cardiovascular cause**	25 (7.3)	18 (5.9)	7 (20)	**0.002**

^ glomerular filtration rate (GFR) estimated by the abbreviated Modification of Diet in Renal Disease (MDRD) equation.

† tHcy, total homocysteine; PML-tHcy, post methionine loading t-Hcy; PML-tHcy increase (i.e. the difference between PML-tHcy and tHcy); the data of PML-tHcy were available for 272 subjects.

Δ LVEF, left ventricular ejection fraction

* values of *P* are calculated by t-test or χ^2^-test.

§ Antiplatelet therapy included Aspirin (80.7%), Ticlopidine (10.8%), and Indobuphen (1.2%).

### Determinants of homocysteine levels

Fasting tHcy levels correlated positively with hs-CRP (*r* = 0.143, *P* = 0.008), and negatively with levels of folate (*r* = −0.330, *P*<0.001), vitamin B12 (*r* = −0.207, *P*<0.001), and estimated GFR (*r* = −0.226, *P*<0.001). The relations with vitamin B6 levels (*r* = −0.097, *P* = 0.077) and with age (*r* = 0.096, *P* = 0.072) were not significant. As expected, *MTHFR 677TT* subjects had increased mean levels of tHcy (20.5 µmol/l, 95% CIs 17.8 to 23.5) as compared to either *677CT* (15.5 µmol/l, 95% CIs 14.7 to 16.2) or *677CC* (14.1 µmol/l, 95% CIs 13.2 to 15.0) subjects (P<0.001 by ANOVA with Tukey post-hoc test). A multiple regression model including all the above mentioned variables showed folate, vitamin B12, GFR, and *MTHFR TT* homozygosity, as independent predictors of tHcy levels (Table 2).

**Table 2:**
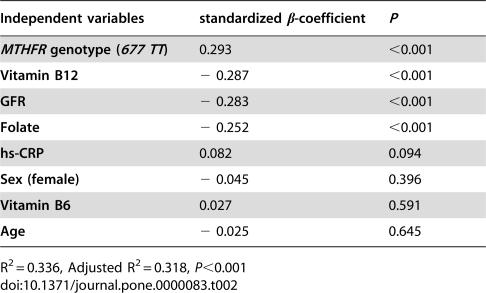
Predictors of tHcy levels (dependent variable) in multiple linear regression analysis.

Independent variables	standardized β-coefficient	*P*
***MTHFR*** ** genotype (** ***677 TT*** **)**	0.293	<0.001
**Vitamin B12**	− 0.287	<0.001
**GFR**	− 0.283	<0.001
**Folate**	− 0.252	<0.001
**hs-CRP**	0.082	0.094
**Sex (female)**	− 0.045	0.396
**Vitamin B6**	0.027	0.591
**Age**	− 0.025	0.645

R^2^ = 0.336, Adjusted R^2^ = 0.318, *P*<0.001

### Mortality by baseline tHcy levels

**Figure 1:**
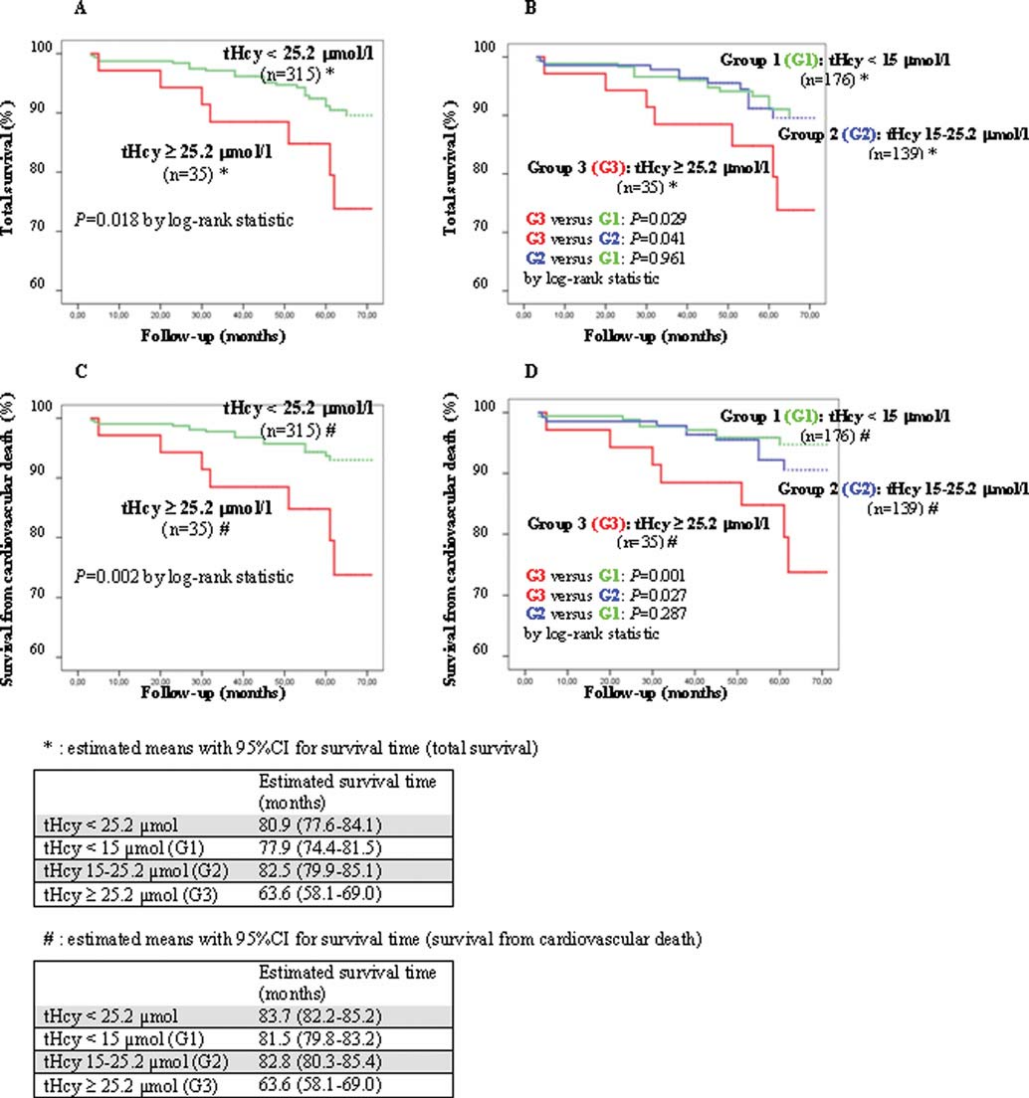
Kaplan-Meier survival plots for total mortality (A–B) and cardiovascular death (C–D) in relation to tHcy levels. Cutoff points for tHcy strata at 25.2 µmol/l in plots A and C, at 15 and 25.2 µmol/l in plots B and D.

After a median follow-up of 58 months (range 3 to 87), 33 patients (9.4%) had died. Twenty-five deaths (75.8% of total deaths) were because of cardiovascular events. The 8 deaths due to noncardiovascular causes were mainly due to cancer (6), one patient died from pneumonia, and one from undetermined cause (he had moved to another country and died there, so that we could not retrieve adequate information). The prevalence of HHcy was significantly higher in total and cardiovascular mortality groups (21.2% and 28.0%, respectively) as compared to survivors (8.8%; *P*<0.05 by χ^2^-test in both cases). In HHcy patients, mortality was nearly twice (20%) as much than in the others, and all deaths in HHcy group were from cardiovascular causes (Table 1). The univariate HRs of HHcy for total and cardiovascular mortality were 2.64, 95% CIs 1.14 to 6.10 (*P* = 0.023), and 3.67, 95% CIs 1.53 to 8.76 (*P* = 0.004), respectively. Figures 1A and 1C represent the Kaplan-Meier survival plots for total and cardiovascular mortality of HHcy patients as compared to the others (log-rank statistic = 5.57, *P* = 0.018; and 9.76, *P* = 0.002, respectively). Since literature often consider a tHcy level of 15 µmol/l as the upper limit beyond which the cardiovascular risk may start to increase [1], we further stratified our population with tHcy<25.2 µmol/l into two subgroups with (*n* = 139) or without (*n* = 176) baseline tHcy levels≥15 µmol/l. However, with respect to either total or cardiovascular (Figures 1B and 1D) mortality, the group with tHcy between 15 and 25.2 µmol/l behave similarly to the group with tHcy <15 µmol/l, and significantly different from that with tHcy≥25.2 µmol/l.

### Other univariate predictors

Statin therapy at discharge was the most powerful prognostic indicator (HRs for total and cardiovascular mortality = 0.14, 95% CIs 0.06 to 0.33; *P*<0.001; and 0.20, 95% CIs 0.08 to 0.50; *P* = 0.001). Other predictive factors in univariate analyses were hs-CRP levels, age, LVEF, and β-blocker therapy at discharge. Details about the relative HRs with 95% CIs are shown in Table 3A, together with values adjusted for age and sex. The *MTHFR 677TT* genotype was not significantly associated to an increased mortality, while we observed a non significant trend toward the contrary: for example, the prevalence of *TT* genotype was only 6.3% in the total mortality group, 8.3% in the group died for cardiovascular causes, as compared to 17.5% in survivors (*P* = NS). Moreover, none of the other determinants of tHcy in the multiple regression model (folate, vitamin B12) showed a predictive value at a *P* level<0.10).

**Table 3A:**
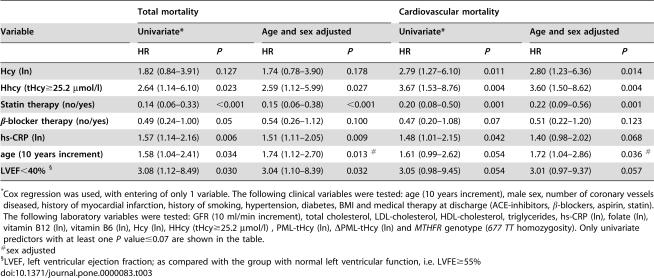
Univariate and sex/age adjusted predictors of total and cardiovascular mortality by Cox regression analyses.

	Total mortality				Cardiovascular mortality			
Variable	Univariate*		Age and sex adjusted		Univariate*		Age and sex adjusted	
	HR	*P*	HR	*P*	HR	*P*	HR	*P*
**Hcy (ln)**	1.82 (0.84–3.91)	0.127	1.74 (0.78–3.90)	0.178	2.79 (1.27–6.10)	0.011	2.80 (1.23–6.36)	0.014
**Hhcy (tHcy≥25.2 µmol/l)**	2.64 (1.14–6.10)	0.023	2.59 (1.12–5.99)	0.027	3.67 (1.53–8.76)	0.004	3.60 (1.50–8.62)	0.004
**Statin therapy (no/yes)**	0.14 (0.06–0.33)	<0.001	0.15 (0.06–0.38)	<0.001	0.20 (0.08–0.50)	0.001	0.22 (0.09–0.56)	0.001
**β-blocker therapy (no/yes)**	0.49 (0.24–1.00)	0.05	0.54 (0.26–1.12)	0.100	0.47 (0.20–1.08)	0.07	0.51 (0.22–1.20)	0.123
**hs-CRP (ln)**	1.57 (1.14–2.16)	0.006	1.51 (1.11–2.05)	0.009	1.48 (1.01–2.15)	0.042	1.40 (0.98–2.02)	0.068
**age (10 years increment)**	1.58 (1.04–2.41)	0.034	1.74 (1.12–2.70)	0.013 #	1.61 (0.99–2.62)	0.054	1.72 (1.04–2.86)	0.036 #
**LVEF<40%** §	3.08 (1.12–8.49)	0.030	3.04 (1.10–8.39)	0.032	3.05 (0.98–9.45)	0.054	3.01 (0.97–9.37)	0.057

* Cox regression was used, with entering of only 1 variable. The following clinical variables were tested: age (10 years increment), male sex, number of coronary vessels diseased, history of myocardial infarction, history of smoking, hypertension, diabetes, BMI and medical therapy at discharge (ACE-inhibitors, β-blockers, aspirin, statin). The following laboratory variables were tested: GFR (10 ml/min increment), total cholesterol, LDL-cholesterol, HDL-cholesterol, triglycerides, hs-CRP (ln), folate (ln), vitamin B12 (ln), vitamin B6 (ln), Hcy (ln), HHcy (tHcy≥25.2 µmol/l) , PML-tHcy (ln), *Δ*PML-tHcy (ln) and *MTHFR* genotype (*677 TT* homozygosity). Only univariate predictors with at least one *P* value≤0.07 are shown in the table.

# sex adjusted

§ LVEF, left ventricular ejection fraction; as compared with the group with normal left ventricular function, i.e. LVFE≥55%

### Multivariate predictive models

Table 3B shows the final multivariate Cox predictive models for total and cardiovascular mortality, obtained by backward stepwise logistic regression approach (*P*≥0.10 to remove). All univariate predictors of total or cardiovascular mortality with *P*<0.10 (Table 3A), including sex, history of myocardial infarction, and all the significant predictors of tHcy (*MTHFR* genotype, glomerular filtration rate, folate, and vitamin B12) were initially entered. Statin therapy was confirmed as the most significant protective predictor. Noteworthy, in multivariate models hs-CRP levels were no longer independent predictors of either total or cardiovascular mortality. Additional stepwise models revealed that this loss of statistical significance was due to adjustment for statin therapy (data not shown). On the contrary, HHcy remained a significant independent predictor even after adjustment for all potential confounders, including major tHcy determinants such as GFR, folate, vitamin B12, and *MTHFR 677 TT* genotype (Table 3B).

**Table 3B:**
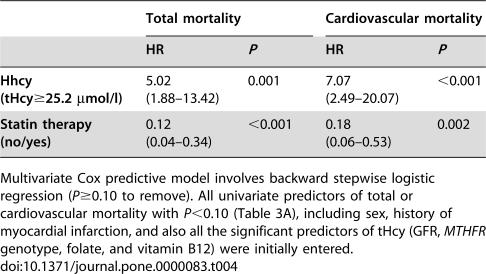
Final Cox predictive models of total and cardiovascular mortality.

	Total mortality		Cardiovascular mortality	
	HR	*P*	HR	*P*
**Hhcy (tHcy≥25.2 µmol/l)**	5.02 (1.88–13.42)	0.001	7.07 (2.49–20.07)	<0.001
**Statin therapy (no/yes)**	0.12 (0.04–0.34)	<0.001	0.18 (0.06–0.53)	0.002

Multivariate Cox predictive model involves backward stepwise logistic regression (*P*≥0.10 to remove). All univariate predictors of total or cardiovascular mortality with *P*<0.10 (Table 3A), including sex, history of myocardial infarction, and also all the significant predictors of tHcy (GFR, *MTHFR* genotype, folate, and vitamin B12) were initially entered.

## Discussion

In this prospective study, hyperhomocysteinemia (>25.2 µmol/l) was an important and independent prognostic factor of mortality after CABG. A particular effort was made to take into account all possible confounders, including the known major genetic (*MTHFR 677C→T* polymorphism) and non-genetic (renal function, B-group vitamin status) determinants of tHcy, as well as modern drug therapy. To put our results into perspective from a clinical standpoint, we propose the following considerations.

### Comparison with previous studies

Most of the prospective studies published so far on tHcy focused on subjects without evident cardiovascular disease at entry, yielding variable results [3], [11]. Relatively few studies [16], [17] focused on patients with angiographically defined CAD, and none of them specifically addressed the prognostic impact of tHcy after CABG. Nygard et al. [16] evaluated mortality in 587 patients with angiographically defined CAD, 318 of them submitted to CABG. After a median follow-up of 4.6 years, they found in the entire population a 10.9% total mortality, with a ratio of 4.5 for patients with the highest (>20 µmol/l) compared to the lowest (<9 µmol/l) tHcy values, after adjustment for traditional risk factors. Anderson et al. [17] studied a total of 1,412 patients after a median follow-up of 3 years from angiography (total deaths: 11.8%), subsequently treated by CABG, angioplasty, or medical therapy alone. In the entire group, subjects with elevated tHcy (mean level 22.9 µmol/l) had a significant mortality risk, but no specific data were given for the CABG subgroup. Our results are well in line with these studies, indicating HHcy as a prognostic marker of mortality in high-risk CAD patients. Moreover, our multivariate adjustment included newer prognostic factors like hs-CRP, major genetic and non-genetic determinant of tHcy levels (*MTHFR* *677C→T* polymorphism, B group vitamins, renal function), and details on current drug therapy after CABG. The predictive value of tHcy remained statistically significant even after correction for a major determinant of mortality such as statin therapy.

The PML test has been suggested as an useful tool to uncover subtle abnormalities of the homocysteine metabolic pathway [18]. In case-control studies, it was reported to identify up to an additional 27% of potentially “at-risk” patients with normal fasting tHcy levels [19]. In the routine clinical setting, however, the value of the PML test has been defined “uncertain” by a recent expert panel [20], which particularly underscored the lack of prospective studies. To the best of our knowledge, this is the first prospective study evaluating the prognostic impact of PML test in CAD patients. Neither PML tHcy absolute values, nor the relative tHcy increase, gave significant predictive information in addition to fasting tHcy. Thus, our data suggest a little value of this cumbersome procedure in the practical scenario.

### MTHFR and risk

Despite being confirmed as a major independent predictor of hyperhomocysteinemia, the *MTHFR 677TT* genotype was not at all a predictor of mortality, even in univariate analysis. Meta-analyses have quantified into ∼2.5 µmol/l the usual *mean* increase in tHcy levels of TT *versus* CC subjects at a population level [21]. As a consequence, several thousands of subjects need to be prospectively followed to see an effect on mortality, which, if present, may indicate a causal link between tHcy and CVD because of the so-called “Mendelian Randomization” [22]. Our study was clearly underpowered for such a purpose, this being likely the main reason for the null finding. On the other hand, we previously demonstrated in this population a substantial gene-environment interaction, so that the *MTHFR 677C→T* polymorphism increased tHcy levels only in individuals with low folate status, while it was neutral in individuals with adequate folate levels [13]. Such an effect was confirmed by a recent meta-analysis [5], revealing that the relation between the *MTHFR* genotypes and CAD, if any exists, is a complex phenomenon whit marked inter-individual variations. Thus, our null finding for *MTHFR* genotypes and mortality is not at all surprising, and in line with the reports from Anderson (mortality in *MTHFR 677TT versus* *677CC* subjects = 11.7% *versus* 15.1%, *P* = 0.62) [17], and from others who observed divergent associations with incident CAD when considering tHcy or the *MTHFR 677C→T* polymorphism [23].

### Other predictors of mortality in post-CABG patients

In this study, statin therapy was the most powerful independent predictor of mortality (Relative Risk Reduction in fully adjusted models: 76–85%). About 60% of our patients, recruited between 1996 and 1999, assumed statin therapy, which was formally introduced as a recommendation into guidelines for CABG surgery only in 1999 [24]. Our data are consistent with previous observations in CABG patients [25]. High-sensitivity CRP, recognized as an important predictor of cardiovascular events in the past few years, was significantly associated to mortality in univariate, and age/sex adjusted, analyses. However, statistical significance was no longer evident after adjustment for statin therapy. This probably reflect the recently highlighted CRP-lowering effect of statins, in a manner largely independent of LDL cholesterol levels [26].

### tHcy and risk: pathopysiological considerations

The association between hyperhomocysteinemia and mortality after CABG represents a clue, but not a proof, of causality. Recently, large randomized trials showed no clinical benefit of tHcy-lowering by means of B-group vitamins in secondary prevention [9], [10]. Of note, however, they enrolled patients irrespective of their baseline tHcy levels, that were generally low (means: 12.2 to 13.2 µmol/l). On the other hand, in our study a well-defined detrimental role of tHcy was evident only for relatively high levels (>25 µmol/l), consistent with those with the highest predictive value on mortality in previous studies on patients with established CAD (see above) [16], [17]. Accordingly, experimental studies on endothelial function in humans suggest that the toxicity of tHcy may be barely discernible at levels only slightly elevated [27]. These considerations may indicate the need of focusing future trials with tHcy-lowering therapy on CAD subjects with more than a slight hyperhomocysteinemia. Nevertheless, it is increasingly recognized that therapy with B-group vitamins interferes with complex phenomena, such as nucleic acid synthesis and DNA methylation [27], [28], suggesting caution, as well as the need of alternative approaches [29].

### Study strength and limitations

A limitation of this study lies on the relatively low number of events. It also implies the limitations of nonrandomized observational studies (i.e., unsuspected selection biases and confounding), and possible inaccuracy in assigning causes of death by certificates. Nevertheless, it has some advantages. First, the clinical homogeneity of the sample (post-CABG patients with advanced atherosclerosis), which, on the other hand, may well represent the real world. Indeed, in terms of age, and burden of classical risk factors (see table 1), our patients fit the typical profile of CAD patients that are seen by cardiologists and cardiosurgeons in daily practice. A second point of strength is the robustness of the endpoint considered, i.e. mortality. By contrast with these points, previous prospective studies on tHcy often included a larger number of CAD patients, but with a wide clinical heterogeneity (different degree of CAD, age at disease onset, interventional or medical therapy), as well as with artificially combined outcomes (cardiovascular “events”, angiographic restenosis, and so on). Further potential advantages of this study are the full adjustment for modern medical treatment and predictors, as well as, for genetic and non-genetic determinants of tHcy, and the absence of confounding from vitamin supplementation. As mentioned above, nowadays the prognostic impact of tHcy may become hard to evaluate in populations such as those from Northern America, where (differently from Europe) folic acid fortification of grain products issued by FDA in 1996 has been demonstrated to effectively reduce tHcy levels [30].

### Conclusions

Irrespective of causality, hyperhomocysteinemia appears as a prognostic marker of mortality after CABG. Its effect was independent of the most significant predictor, which was represented by the lipid-lowering, and probably anti-inflammatory, therapy with statins. Measuring tHcy before CABG may help to identify a high-risk subgroup of patients for whom it may be especially important to follow a healthy lifestyle and diet, as well as to receive optimal treatments for known causal risk factors. Further studies with adequate design are needed to clarify whether or not patients with HHcy should take advantage from tHcy-lowering therapy, as well as to identify the tHcy threshold level that would be worth to treat, to reduce mortality after CABG.
